# Impact of health investment on household income distribution: insights from China’s longitudinal survey data

**DOI:** 10.3389/fpubh.2024.1346133

**Published:** 2024-04-08

**Authors:** Lili Zheng, Wenhua Hou, Ming Huang

**Affiliations:** ^1^School of Insurance, Central University of Finance and Economics, Beijing, China; ^2^Medical Department, Aviation General Hospital, Beijing, China

**Keywords:** health investment, income distribution, China nutrition and health survey, urban and rural disparities, income variance, income mobility

## Abstract

This paper investigates the impact of health investment on household income distribution, drawing from data spanning over 10 years from the China Nutrition and Health Survey. The study aims to contribute to the literature by examining the nuanced pathways through which health investment influences income distribution. Utilizing a rich dataset, rigorous empirical methods including quantile regression and cross-sectional data modeling are employed to explore the relationship between health investment and income distribution. The analysis reveals a robust positive association between health investment and both absolute and relative income levels across various demographic and occupational groups. Additionally, the study elucidates the pathways through which health investment influences income, including its effects on illness duration, employment opportunities, effective working time, and educational attainment. The findings demonstrate the dynamic nature of the relationship, indicating that as income levels rise, the impact of health investment on income becomes more pronounced. Moreover, the analysis highlights the role of health investment in facilitating upward income mobility, particularly for low-income households. Overall, these findings provide valuable insights for policymakers, suggesting that strategic health investment initiatives can contribute to achieving more equitable income distribution.

## 1 Introduction

The role of health in income distribution is of paramount importance, with historical precedents emphasizing health as a vital component of human capital development (1). Additionally, endogenous growth theory has highlighted health as a crucial driver of economic development, often surpassing the influence of other factors (2). The economic ramifications of health are substantial, as underscored by the American Cancer Society’s report in 2009, which estimated that cancer alone led to economic losses of $895 billion in 2008, equivalent to 1.5% of the global GDP (American Cancer Society, 2009). These losses are primarily attributed to disability resulting from illness and decreased work productivity. The impact extends further to opportunity costs, encompassing the net present value of future benefits forfeited due to premature deaths, lost working hours, and medical expenses. The interplay between health and economic growth has garnered considerable attention within the economics community. From a microeconomic standpoint, health can be integrated into an individual’s utility function, signifying it as both a consumable good and an investment with a favorable return (2–4).

Recent studies underscore the significant economic benefits of investing in health. A larger and healthier labor force is associated with substantial economic benefits across countries, as healthy workers are more productive and contribute more effectively to the economy (Brookings Institution, 2021). Public health expenditure in the United States is linked to improved economic performance because it strengthens human capital and increases productivity (5). In the realm of economic growth theory, health assumes a dual role: as an intrinsic value, directly benefiting personal well-being and societal advancement, and as a tool, contributing instrumentally to various facets of human development, including income augmentation (3–8). Research has demonstrated a dynamic relationship between health improvement and economic growth, forming a ‘virtuous spiral’ where health advancement fosters economic prosperity, and vice versa, transforming nations from low-income and disease-prone states into high-quality life providers for their citizens (3, 9).

Conversely, the detrimental economic impact of declining health is starkly evident in the case of Russia, where a substantial premature death toll between 1990 and 1995, exacerbated economic instability and income declines, propelling a vicious spiral (10). More recently, the COVID-19 pandemic in 2020 inflicted a $6.6 trillion cost on the global economy (10). Human capital arising from health investment features unique production functions and stands as an essential prerequisite and input in the production process. It impacts individual labor productivity, work duration, and education investment returns, thereby influencing labor supply through mortality and life expectancy adjustments, ultimately affecting the production function and, consequently, income (3, 6, 8).

Despite the extensive research on the topic, gaps remain in our understanding of the complex interplay between health and the economy, rendering the analysis of the multifaceted economic and social pathways challenging. Additionally, establishing causal relationships in the context of economic prosperity promoting health, demographic factors, technological advancements, and institutional enhancements pose further theoretical and empirical challenges (3, 9). Building on the existing body of research, this paper defines health investment as the allocation of resources toward the physical and mental well-being of individuals, encompassing elements such as consumption, nutrition, medical care, physical activity, and living environment improvement (China Nutrition and Health Survey, 1989–2015). The study explores the income effect of health investment from a microeconomic perspective (China Nutrition and Health Survey, 1989–2015).

The innovation of this article can be identified in two key aspects. Firstly, it addresses a gap in prior research by shifting the focus from analyzing the income distribution effects of health investment solely from the perspective of medical financing to a broader examination of health investment itself. Previous studies often relied on health indicators that exhibit heterogeneity due to various factors such as investment behavior, genetic influences, random shocks, and measurement errors (11). These multifaceted health indicators do not always align with traditional dimensions like mortality, morbidity, and quality of life. Consequently, there is a lack of consensus among researchers on how to accurately define and measure health at the individual level, leading to challenges in depicting time series analyses of health impacts. By examining the impact of health investment on income, this paper circumvents these challenges. It treats health investment as a cross-sectional indicator of individuals’ sustained health or health changes over time, offering an improved ability to distinguish chronic health issues from acute, short-term interventions. This approach posits that health investment indicators provide more accurate insights compared to traditional health indicators utilized in previous studies.

Secondly, this paper delves into the bidirectional relationship between health investment and income, a dimension often overlooked in existing literature. It acknowledges that health holds both “consumer value” and unique “capabilities, “wherein healthier individuals tend to have higher incomes, and vice versa. However, the intricate nature of this bidirectional relationship has led to a lack of consensus in evaluating the economic impact of health policies. The assessment of overall policy priorities and the relative effectiveness of specific categories of health expenditures often relies on expert “best judgment” (12). The interaction between health and income is typically estimated directly through health reduction models or income equations (13). However, when adopting the simultaneous equation framework, the estimation of the relationship between health and income heavily depends on potential identification assumptions (14). The bidirectional connection between health investment and income presents a pivotal challenge for empirical research. This study accounts for the income situation in the health investment equation and addresses the endogeneity of health status in the income equation. It employs a two-stage model as the benchmark, examining the relationship between health investment and income. Initially, the impact of health investment on health is assessed, followed by an evaluation of the effect of health on income. This design allows for tracing the influence of health investment on economic welfare, considering both the direct impact of poor health on income and the indirect influence through variables such as illness duration, employment, effective labor time, and education. To enhance the reliability of the results, the benchmark model adjusts for sample selectivity and controls for factors influencing income to minimize the risk of biased findings. The heterogeneity test clarifies the impact of health investment on income among different groups, while the robustness test section explores alternative methods, including quantile regression and cross-sectional data models. The variable selection incorporates measures of absolute income, relative income, and income liquidity, offering a comprehensive examination of the impact of health investment on income from multiple perspectives.

## 2 Literature review

The connection between income and health has been extensively studied, revealing a strong correlation where higher income enables access to health-promoting resources such as quality nutrition and healthcare [e.g., (15–20)]. While most research has focused on how income influences health, another dimension explores the causal link from health to income. Scholars widely agree that improvements in health positively impact the economy, as evidenced by the significant role of health in driving economic success, particularly in East Asia (2, 21–27). Conversely, poor health can hinder economic progress, altering population structures and creating poverty traps, especially in regions like Africa (28).

Luft (29) highlighted the significant impact of poor health on income, demonstrating a marked reduction in annual earnings for individuals with disabilities. This relationship between health and income is further supported by numerous longitudinal studies [e.g., (26, 30–32)]. The complexity of the health-income interplay extends to factors such as labor participation, employment duration, unemployment rates, working hours, and wages. Health emerges as a key determinant of labor force participation, as supported by various economic models that incorporate health status [e.g., (14, 24, 33–37)].

Moreover, health investments play a crucial role in shaping income, with nutrition and body mass index (BMI) emerging as significant predictors of productivity (38). Interventions aimed at improving health, particularly for women and children, have been linked to economic growth (39, 40), with studies by Strauss (41) and others (42–45) reinforcing the idea that health investments enhance income. Hamid et al. (46) identified health’s dual impact on income—the “enhancement effect” and the “stabilization effect”–suggesting that healthier individuals not only translate human capital into sustainable income but also reduce healthcare expenses by increasing employment opportunities and working hours.

The economic impact of health investments operates through several key mechanisms. Firstly, a positive correlation between healthcare expenditure and labor productivity suggests that healthier individuals are more energetic and capable, thereby enhancing productivity and income (5). Secondly, the intersection of health and education investments yields significant returns, with women’s health playing a pivotal role in the long-term productivity and economic performance of nations (5). This interplay suggests that healthier individuals, anticipating a longer life, are more likely to pursue educational opportunities, thus bolstering their skill sets and income potential.

Moreover, the direct economic benefits of health investments are evident in labor market outcomes; for instance, the NHS Confederation reports a quadruple return on every pound invested in health through improved productivity and labor participation. Corporate health programs have also shown to offer a 47% average return on investment, underscoring the financial viability of investing in employee health (47). Lastly, improved health outcomes are associated with extended lifespans, prompting individuals to save more for retirement, thus leading to increased capital accumulation and income growth.

Furthermore, investment in health is posited to drive overall economic development through the accumulation of human and physical capital (48). Studies suggest that for every dollar invested in better health, an economic return of $2 to $4 could be realized, highlighting the cost-effectiveness of health investments (49). Additionally, investment in health data is identified as a driver of economic growth, especially in low-and middle-income countries, as it can lead to development and improve disease management (50).

In conclusion, the findings collectively illustrate that health investment is not only a catalyst for enhancing individual income but also a strategic component in fostering broader economic growth and development.

## 3 Data, variables, and model design headings

### 3.1 Data source

The dataset utilized in this study originates from the China Health and Nutrition Survey (CHNS), a longitudinal survey jointly conducted by the University of North Carolina and the Chinese Preventive Medicine Association in the United States. Representing one of the few longitudinal panel tracking surveys in China, the CHNS spans a significant period, comprising 10 follow-up surveys conducted in 1989, 1991, 1993, 1997, 2000, 2004, 2006, 2009, 2011, and 2015. The survey encompasses a diverse sample of approximately 7,200 households and over 30,000 individuals across 15 provinces and municipal cities, capturing variations in geography, economic development, public resources, and health indicators. Notably, the CHNS survey engages multiple disciplines, including food markets, health facilities, family planning officials, as well as other social services and community leaders. This comprehensive approach ensures a holistic understanding of health and nutrition trends over time, facilitating nuanced analyses of the interplay between health investment and household income distribution.

We use survey data from 10 rounds of CHNSs in 1989, 1991, 1993, 1997, 2000, 2004, 2006, 2009, 2011, and 2015 to conduct research. We select the years 1989 and 2015 as primary data points, with the intervening years serving as supplementary. The years 1989 and 2015 represent the start and the most recent data collection points of the CHNS, allowing for a comprehensive longitudinal analysis. This period covers significant socio-economic transitions in China, providing a unique opportunity to study the impacts of these changes over a generation. This longitudinal approach is vital for understanding how changes over time are associated with various outcomes, offering insights that cross-sectional studies cannot provide. By leveraging the full spectrum of data from the CHNS, we can conduct a more nuanced and detailed analysis. This approach allows for the examination of intermediate effects, the impact of specific policies or global events, and the interaction between various factors over time. Although the survey extends up to 2015, it also provides a robust and comprehensive dataset that allows for a thorough examination of long-term trends and patterns in health investment and income distribution. By analyzing the trends observed up to 2015 in conjunction with existing knowledge of more recent developments in healthcare policies, socioeconomic conditions, and other relevant factors, we can infer potential trajectories and anticipate the impact of recent trends or policy changes on health investment and income distribution.

To ensure the integrity of the analysis, a meticulous data cleaning process was undertaken. Firstly, individuals under the age of 18 were excluded from the sample, as were retired seniors, the latter to minimize potential distortions arising from retirement decisions on income dynamics (34). Additionally, rigorous measures were taken to identify and remove samples exhibiting significant missing data and outliers. Despite these exclusions and occasional gaps in certain health investment variables across specific years, the resulting dataset comprised a robust set of 79,715 observations drawn from 10,802 individuals, spanning a comprehensive 10-year period. This refined dataset forms the foundation for the subsequent analyses, ensuring the reliability and validity of the study findings.^1^

### 3.2 Variables

For variable selection, income serves as the dependent variable in two forms: absolute and relative. Absolute income is quantified as monthly salary, while objective relative income is gaged using a ratio–inspired by Ravallion and Lokshin (51) and Powdthavee (52)–of the household’s *per capita* income to the median *per capita* income within their city, providing a measure of relative wealth.

The CHNS data includes a comprehensive set of adult nutrition and health status indicators. Following Zheng (53), we select variables of health investment such as nutrition, medical care, physical activity, and living conditions, employing factor analysis to construct a composite health investment index.

In line with established literature (51, 52, 54), the study incorporates control variables that may influence income, including both demographic-sociological and socio-economic characteristics. Demographic variables encompass gender, registered residence, age, education level, and marital status. Economic variables of households include employment status, property ownership, and material assets, providing a multidimensional view of the factors contributing to income levels (Table 1).

**Table 1:** Variable design.

Variable classification	Variable measurement	Variable name	Variable description
Dependent variable	Absolute income	Income	Monthly salary
	Relative income	Reincome	the ratio of *per capita* income of sample households to the median *per capita* income of households in the city where the sample is located
Independent variable	Hinvest	Coinvest	Including *per capita* calorie intake, *per capita* protein intake, *per capita* fat intake, and *per capita* carbohydrate intake
		Medinvest	Including smoking, drinking, medical insurance, utilization of medical services, and preventive healthcare
		Spoinvest	Exercise status and sleep time
		Envinvest	Drinking water sources, toilets
Control variable	Sociological characteristics of population	Gender	0 = male, 1 = female
		urban	0 = urban, 1 = rural
		Age	continuous variable
		Education	0 = not attended primary school, 1 = primary school education, 2 = junior high school education, 3 = high school education, 4 = vocational and technical education, 5 = university education, 6 = master’s degree or above
		Marry	Virtual variable, 0 = unmarried, divorced, widowed, separated, 1 = married
	Socioeconomic characteristics	job	Virtual variable, 0 = no, 1 = yes
		House	Current value of family housing and real estate
		Assets	The proportion of the number of household appliances owned by the family and the proportion of the number of means of transportation owned

### 3.3 Benchmark model

The interrelation between health investment and income is complicated by the potential for causal endogeneity. When measurement errors in health status indicators arise from heterogeneous processes, it becomes critical to treat health variables as endogenous in our analyses. To address this issue, this study employs the use of instrumental variables (IVs) to estimate their effects on income. The two-stage least squares (2SLS) model is particularly adept at handling the endogeneity problem, accounting for the correlation between error terms and mitigating the biases associated with the choice of IVs (55). Consequently, this paper adopts the 2SLS model, incorporating binary health knowledge as the IV for the two-stage regression. The rationale behind this selection lies in the fact that while health knowledge exerts a positive influence on individual health investment, its correlation with the error term is minimal, thereby providing a robust solution to the issue of sample self-selection. The first phase of estimation will apply this framework.^2^


(1) $$hinvest_{it}=\alpha_{0}+\alpha_{1}Dietary_{it}+\overset{N}{\underset{k=1}{\sum }}\alpha_{k}X_{kit}+\varepsilon_{it}$$

In the model (Eq. 1), $hinvest_{it}$ serves as the dependent variable, encapsulating the overall health investment of an individual. $Dietary_{it}$ denotes the individual’s health knowledge, which is a critical factor in their health investment decisions. $X_{kit}$ represents the control variables that may influence health investment; these include measures of health status such as short-term health evaluations and long-term health conditions, which are incorporated in the first stage of the model. $\varepsilon_{it}\;$ is the stochastic error term, capturing the random fluctuations that cannot be explained by the model.

The subsequent step involves utilizing the ordinary least squares (OLS) model to ascertain the effects of health investment on health outcomes. The model’s specific functional form is prepared to be applied in this analysis.


(2) $$Income_{it}=\beta_{0}+\beta_{1}hinvest_{it}+\overset{N}{\underset{k=1}{\sum }}\beta_{k}X_{kit}+\mu_{it}$$

In the model (Eq. 2), $Income_{it}$ is the dependent variable, representing the income variable; $h{\mathrm{invest}}_{it}$ is the health investment variable; $X_{kit}$ is a set of control variables, and $\mu_{it}$ is a random disturbance term (Table 2).

**Table 2:** Descriptive statistics.

variable	Mean	Variance	Minimum	Maximum	Sample size
Income	1351.488	1611.984	100.000	10000.000	22427.000
Reincome	0.513	0.286	0.010	0.990	22427.000
Hinvest	-0.004	0.227	−0.68264	0.646	36616.000
Gender	0.512	0.500	0.000	1.000	79715.000
Urban	0.689	0.463	0.000	1.000	79715.000
Age	41.866	13.137	18.000	65.000	79715.000
Education	1.840	1.369	0.000	5.000	77039.000
Marry	0.816	0.388	0.000	1.000	79715.000
Job	0.731	0.443	0.000	1.000	78650.000
House	170143.000	454787.100	0.000	3000000.000	44402.000

## 4 Empirical research

### 4.1 Descriptive statistical results

The descriptive statistics presented in the table offer a detailed snapshot of the study’s socio-economic and demographic variables. The average income among the 22,427 respondents is 1,351.488, with a variance that underscores substantial income disparity. In contrast, the relative income is more consistent, with an average of 0.513, indicating that the typical household earns about half the median city income. The health investment score has a marginal average of−0.004, but the variance suggests a range of health investment practices among the 36,616 individuals analyzed.

### 4.2 The impact of health investment on income

We do a series of diagnostic tests to the instrumental variables within the model to ensure their robustness and appropriateness. These tests–under-identification, weak instrumental variables, and over-identification–affirmed that the chosen instruments are neither weak nor redundant. Following this validation, the two-stage least squares (2SLS) model, incorporating these instrumental variables, was employed to examine the influence of health investment on income (Table 3).

**Table 3:** Health investment and income: 2SLS regression with instrumental variables.

	(1)		(2)	
variable	One stage regression (Dependent variable: coinvest)	Two stage regression (Dependent variable: income)	One stage regression (Dependent variable: coinvest)	Two stage regression (Dependent variable: income)
Score		2.202***		2.834***
		(2.645)		(2.953)
Dietary	0.017**		0.016**	
	(2.095)		(2.050)	
Sick	−0.020		−0.018	
	(−1.245)		(−1.130)	
Chro	0.039***		0.039***	
	(2.885)		(2.881)	
Gender	0.035***	−0.373***	0.035***	0.051
	(4.853)	(−10.100)	(4.838)	(1.202)
Urban	−0.024***	−0.031***	−0.025***	−0.112***
	(−3.361)	(−2.994)	(−3.479)	(−3.032)
Age	−0.001***	0.003**	−0.001***	0.006***
	(−3.477)	(2.111)	(−3.560)	(3.600)
Education	0.017***	0.096***	0.017***	0.097***
	(6.256)	(5.443)	(6.346)	(4.725)
Mary	−0.013	0.181***	−0.013	0.034
	(−1.188)	(4.742)	(−1.124)	(0.778)
House	0.020***	0.057***	0.020***	0.006
	(7.496)	(2.863)	(7.176)	(0.263)
Assets	0.025**	0.005	0.026**	0.033
	(2.087)	(0.100)	(2.159)	(0.608)
constant	−0.187***	6.081***	−0.181***	2.180***
	(−4.560)	(26.930)	(−4.371)	(8.611)
*N*	4,803	4,803	4,761	4,761

The values in parentheses are *t*-values, where *represents a 10% significance level, **represents a 5% significance level, and ***represents a 1% significance level.

The analysis of the two-stage model yields significant insights. In the first stage, health knowledge—an instrumental variable–is shown to have a positive and statistically significant effect on health investment at the 5% confidence level. The second stage of the model reveals that health investment positively influences income, substantiating the hypothesis that health investment is a crucial determinant of both absolute and relative income. This finding is in line with the research of Strauss and Thomas (2) and Lorentzen et al. (23), which also documented the link between health and income.

### 4.3 Heterogeneity testing

The subsequent inquiry addresses whether the income effects of health investment vary across urban and rural locales, different regions, and among various professions. Exploring these distinctions is crucial for enhancing income distribution and optimizing the allocation of health investments. To this end, we will undertake heterogeneity testing to gain a deeper understanding of these dynamics and inform more equitable and effective policy decisions.

#### 4.3.1 Urban–rural differences in the income effect of health investment

In the context of China’s dual economic system, rural residents often encounter systemic challenges that impede income growth. Hindered by the human capital cost premium and labor transfer barriers stemming from the household registration system, they face a more stringent income accumulation mechanism compared to their urban counterparts, making their ascent to the middle-income bracket more arduous. Prior research, such as the work of Zhang and Zhao (56), has highlighted that health’s role in the urban–rural income disparity is significant. Therefore, it is imperative to scrutinize the differential impact of health investment on income between urban and rural populations to address these disparities effectively (Table 4).

**Table 4:** Urban–rural differences in the income effect of health investment.

	Dependent variable: income			
variable	Urban areas	Rural areas	Urban areas	Rural areas
Score	1.606**	1.718**	1.136**	1.565***
	(2.263)	(2.461)	(2.092)	(2.642)
control variable	Join	Join	Join	Join
Year fixed	Join	Join	Join	Join
Family fixed	Join	Join	Join	Join
N	2,152	2,651	2,152	2,651

The values in parentheses are *t*-values, where *represents a 10% significance level, **represents a 5% significance level, and ***represents a 1% significance level.

The model’s outcomes suggest that health investment has a more pronounced effect on the incomes of rural populations compared to urban ones, implying that such investments are instrumental in bridging the urban–rural income divide. For rural residents, who often depend on physical labor, health is a critical determinant of income. Illnesses can prevent engagement in productive work, causing an immediate reduction in earnings. Chronic or severe diseases, particularly those with prolonged recovery times, can lead to sustained decreases in household income. Consequently, health investment carries more weight for rural residents, affecting their economic well-being significantly more than it does for urban dwellers.

#### 4.3.2 Regional differences in the income effect of health investment

China exhibits regional disparities in economic development and health levels, necessitating an examination of how these differences influence the income effects of health investment. In accordance with the classification by the National Bureau of Statistics of China, the country is partitioned into three main economic regions: eastern, central, and western. The CHNS data includes several provinces within these regions: Beijing, Shanghai, Shandong, Jiangsu, and Liaoning in the east; Henan, Hubei, Hunan, and Heilongjiang in the central region; and Guangxi and Guizhou in the west. To address the regional variations, this study segments the overall sample into three distinct sub-samples corresponding to these regions. Each sub-sample is then analyzed individually to assess the regional impact of health investment on income, providing a nuanced understanding of the interplay between regional economic conditions, health investment, and income (Table 5).

**Table 5:** Regional differences in the income effect of health investment.

	Dependent variable: income			Dependent variable: reincome		
Variable	Eastern part	Middle part	West part	Eastern part	Middle part	West part
Score	4.345***	2.770***	1.921**	4.688***	0.193	0.469**
	(5.496)	(3.963)	(2.517)	(5.346)	(0.984)	(2.496)
control variable	Join	Join	Join	Join	Join	Join
Year fixed	Join	Join	Join	Join	Join	Join
Family fixed	Join	Join	Join	Join	Join	Join
N	2,302	1,526	794	2,302	1,526	794

The values in parentheses are *t*-values, where *represents a 10% significance level, **represents a 5% significance level, and ***represents a 1% significance level.

Upon analyzing the influence of health investment on income across the eastern, central, and western regions, it is evident that health investment significantly affects absolute income at both the 5 and 1% confidence levels. Notably, the eastern region, which boasts higher levels of health investment and absolute income, also experiences a more substantial impact from health investment on absolute income. Furthermore, the effect of health investment on relative income is significant at the 5 and 1% confidence levels. When compared to the western region, the eastern region shows a greater degree of impact on relative income, indicating regional disparities in the benefits accrued from health investments.

#### 4.3.3 Job differences in the impact of health investment on income

Zang and Bai (57) contend that employment in the state-owned sector can enhance both personal and family incomes, whereas individuals in the non-state-owned sectors experience income and status fluctuations that are heavily influenced by the macroeconomic climate and policy shifts, leading to instability. Similarly, Yang and Li (58) observed that employment within state-owned enterprises is linked to a favorable upward mobility in income, and working in the secondary industry also tends to facilitate upward income movement. With these perspectives in mind, the forthcoming analysis will categorize the impact of health investment on income based on enterprise type within the sample. The first category encompasses government agencies, state-owned commercial units, and state-owned enterprises. The second includes small and large collective enterprises, along with foreign-funded enterprises. The third category comprises household contract agriculture and private or individual businesses, allowing for a nuanced understanding of health investment’s influence across different industrial sectors.

Table 6 show that entities such as government agencies, state-owned commercial units, and state-owned enterprises experience a more substantial impact on absolute income from health investments. In contrast, sectors like household contract agriculture, along with private and individual enterprises, see a less pronounced effect on absolute income. Similarly, the impact of health investment on relative income is significant at the 1% confidence level, with state-owned entities again showing a greater influence, while the private sector, including household agriculture and individual enterprises, demonstrates a comparatively lesser impact on relative income.

**Table 6:** Job differences in the income effect of health investment.

	Dependent variable: income			Dependent variable: reincome		
variable	Category 1	Category 2	Category 3	Category 1	Category 2	Category 3
Score	9.206***	4.155***	2.931***	7.764***	3.248***	0.822***
	(2.670)	(3.993)	(5.965)	(2.578)	(3.190)	(2.863)
control variable	Join	Join	Join	Join	Join	Join
Year fixed	Join	Join	Join	Join	Join	Join
Family fixed	Join	Join	Join	Join	Join	Join
N	1918	601	2,124	1918	601	2,124

The values in parentheses are *t*-values, where *represents a 10% significance level, **represents a 5% significance level, and ***represents a 1% significance level.

### 4.4 Mechanism of the impact of health investment on income

The preceding analysis suggests that health investment impacts income through a multitude of channels, warranting a detailed examination of the underlying mechanisms. This article draws upon existing literature to dissect four primary pathways:

Firstly, health investment influences the duration and severity of illness, where robust physical fitness and swift recovery can mitigate direct economic losses incurred by disease. Investments in health can diminish the risk and repercussions of illness for individuals and families, curtailing the time and opportunity costs associated with medical treatment and work absences, thus bolstering individual income.

Secondly, education plays a pivotal role. Empirical evidence by Wolf (59) demonstrates that poor health can truncate educational attainment and erode the quality of schooling, thereby reducing income potential. Conversely, individuals in good health are more likely to pursue further education and training, amassing human capital that translates into higher earnings. Moreover, health promotes a longer life expectancy, influencing family decisions on education investment. As Schultz (13) observed, a sound health level encourages workers to seek additional education and training to enhance their skills and, consequently, their income.

Thirdly, employment opportunities are significantly swayed by health. A robust health status opens more job opportunities, as noted by Luft (29). Studies by Schultz and Tansel (21), as well as research based on US data by Fan (60), support the assertion that health positively impacts employment. Wei (25) furthers this, emphasizing the influence of health on labor participation and opportunities outside of agriculture.

Fourthly, effective working time is a crucial factor. Personal health can improve work attendance, reduce absenteeism, and enhance overall work efficiency. Health investment positively affects the physical and mental well-being of workers, enabling them to meet the demands of high-intensity labor and increasing productivity, which is advantageous for income levels (2).

To analyze the pathways through which health investment affects income, this article employs a structural equation model to construct a path framework diagram, testing the effects of health investment on income via these four pivotal variables.

**Figure 1:**
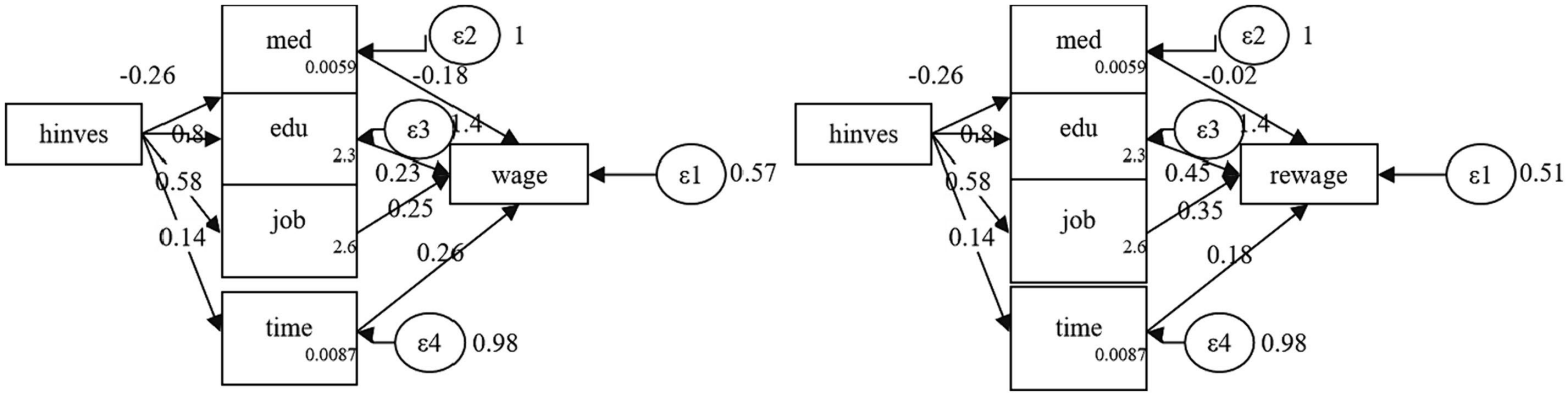
Mechanism of the impact of health investment on income. In this figure presents a conceptual framework illustrating the pathways through which health investments may influence income. Within this structural model, health investments are postulated to exert both direct and indirect effects on income. Notably, there exists a negative correlation between health investment and the duration of illness (“med”), suggesting that increased health investments are associated with shorter periods of illness. Moreover, the model illustrates that health investments positively impact education (“edu”), subsequently leading to higher income levels (“wage” in this Figure and “rewage” in Figure 2). This implies that individuals with better health may attain higher levels of education, thereby enhancing their earning potential. Additionally, a positive relationship exists between health investments and the probability of employment (“job”), mediated through a Bernoulli distribution, indicating that greater health investments may augment the likelihood of securing employment. Furthermore, health investments positively influence effective labor time (“time”), suggesting that healthier individuals may engage in longer work periods, potentially resulting in increased income. While the indirect effects of health investment through these pathways are relatively smaller compared to its direct impact on income, they remain significant. In both figures, “wage” and “rewage” represent distinct measures of income, while factors such as education, employment, and effective labor time positively influence the income variable. Each endogenous variable in the model–med, edu, job, and time–is associated with an error term (ε2, ε3, ε4), accounting for unexplained variation. Additionally, the income variable includes a residual term (ε1), representing unexplained variability in income. Although the models depicted in the figures are similar, slight variations in the income variable and coefficients associated with the pathways may arise from differences in income operationalization, sample characteristics, or modeling conditions. Overall, these figures underscore the multifaceted impact of health investments, not only in mitigating illness but also in fostering broader socioeconomic advantages such as improved education outcomes, enhanced employment prospects, and increased labor productivity, all of which contribute to higher income levels.

Figure 1 illustrates the multifaceted effects of health investment. It demonstrates a negative correlation between health investment and the duration of illness, indicating that greater health investment is associated with shorter illness durations. Conversely, the impact of health investment on education, employment, and effective labor time is positive, suggesting that such investment enhances these factors. The duration of illness has a negative effect on both absolute and relative income, whereas education, employment, and effective labor time all have a positive influence on income levels. While the indirect effects of health investment through these pathways are smaller compared to its direct effect on income, the influence is nonetheless significant. Health investment can meaningfully increase both absolute and relative income through its effects on the duration of illness, educational attainment, employment opportunities, and effective labor time.

## 5 Robustness testing

### 5.1 Quantile regression

Quantile regression is employed to assess the marginal contribution of health investment to individual income across different income brackets. This method is adept at minimizing the asymmetry of absolute residuals and incorporates instrumental variables to enhance the robustness of the estimates. The crux of the quantile analysis is to explore variations in the income effects of health investment across various income levels.

**Figure 2:**
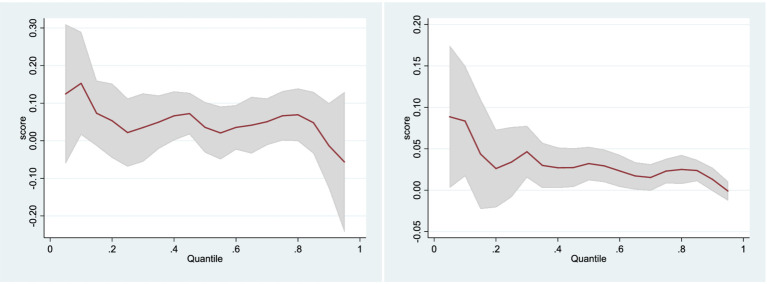
Quantile regression with absolute income and relative income. Figure 1 illustrates the nuanced effects of health investment on absolute income across different levels of income distribution, as determined by the quantile regression analysis. The red line represents a positive effect on income at lower quantiles, indicating that health investment is particularly advantageous for individuals with lower incomes. However, this effect gradually diminishes and even sharply declines beyond the 80th percentile, suggesting diminishing returns for higher-income individuals. The widening confidence intervals observed at higher quantiles signify increased uncertainty regarding these effects among individuals at the upper end of the income spectrum. In this Figure, focusing on relative income, demonstrates notable variations in the impact of health investment across different income groups. A significant positive effect is evident at lower quantiles, particularly around the 30th percentile, suggesting that lower earners derive greater relative income benefits from health investment compared to their counterparts. However, this effect diminishes beyond the 40th percentile, with a downward trend indicating reduced benefits for individuals above the median income level. Similar to the previous figure, the varying width of the confidence intervals across different income levels underscores the variable certainty regarding the estimated effects of health investment.

Figure 2 reveals that health investment notably enhances individual absolute income, yet this income effect diminishes as one moves up the income ladder, with a marked decline at the 80th percentile where the utility derived from income sharply tapers off. The figure also indicates that health investment considerably boosts individual relative income, with the impact varying significantly across income groups. For individuals at the lower end of the relative income spectrum, the benefits of health investment are relatively more pronounced, exhibiting notable fluctuations around the 30% quantile. Beyond the 40% quantile, the positive impact of health investment on income progressively lessens.

### 5.2 Cross section data inspection

To examine the time-specific effects of health investment on income, the analysis employs cross-sectional data exclusively from the year 2006, which serves as a robustness check for the observed temporal impact. Selecting cross-sectional data in 2006 because it offered the largest sample size among available datasets. By choosing data from 2006, we aimed to maximize the representativeness and statistical power of their analysis by leveraging a larger and more diverse sample, allowing for a more robust examination of the variables and relationships under investigation (Table 7).

**Table 7:** Robustness test of cross section data.

variable	Dependent variable: income	Dependent variable: reincome
Hinvest	0.314**	0.111**
	(1.989)	(2.298)
Control variable	Join	Join
Year fixed	Join	Join
Family fixed	Join	Join
*N*	2,206	2,651

The values in parentheses are *t*-values, where *represents a 10% significance level, **represents a 5% significance level, and ***represents a 1% significance level.

The analysis of the 2006 cross-sectional data yields significant results: health investment has a discernible impact on both absolute and relative income at a 5% confidence level. This finding reinforces the robustness of the earlier results, confirming the substantial influence of health investment on income.

### 5.3 The dynamic impact of health investment on income

Health investment elevates individual income levels have been demonstrated, however, questions remain about the long-term sustainability of this effect. What is the dynamic impact of health investment on income over time? Addressing these questions is crucial to understanding when health investment influences income and to ascertain the enduring nature of its effects. To delve into these issues, we construct the following econometric model:


(3) $$\begin{array}{l} Income_{it}=\beta_{0}+\beta_{1}\overset{N}{\underset{k=1}{\sum }}\lambda_{\tau }hinvest_{it-\tau }+\beta_{2}IV_{it}+\\ \overset{N}{\underset{k=1}{\sum }}\beta_{k}X_{it}+\lambda_{i}+\varepsilon_{it} \end{array}$$

In the model (Eq. 3), $\lambda_{i}$ is the number of lag periods ( i =0,1,2,3), the estimated coefficient $\lambda_{\tau }$ depicts the dynamic impact of health investment on income (Table 8).

**Table 8:** Dynamic impact of health investment on income.

	Dependent variable: Absolute income				Dependent variable: Relative income			
Hinvest	2.202***				2.834***			
	(2.645)				(2.953)			
Hinvest(-1)		1.565^*^				0.691***		
		(1.830)				(2.927)		
Hinvest(-2)			1.321***				0.297***	
			(2.701)				(2.589)	
Hinvest(-3)				0.689				0.031
				(0.906)				(0.173)
Control variable	Join	Join	Join	Join	Join	Join	Join	Join
Year fixed	Join	Join	Join	Join	Join	Join	Join	Join
Family fixed	Join	Join	Join	Join	Join	Join	Join	Join
Instrumental variable	Join	Join	Join	Join	Join	Join	Join	Join
sample size	4,761	1,393	1,128	1907	4,761	1,393	1,128	1907

The values in parentheses are *t*-values, where *represents a 10% significance level, **represents a 5% significance level, and ***represents a 1% significance level.

The model’s estimation results, presented in Table 6, reveal that the coefficients for health investment across the current period, as well as lagged periods 1, 2, and 3, are all positive. The coefficients for the current period and the first two lagged periods are statistically significant, with the magnitude of these coefficients exhibiting a declining trend. This pattern suggests that while the influence of health investment on income diminishes over time, the positive effect it exerts is sustained, affirming the enduring promotive role of health investment on income levels.

## 6 Research based on income liquidity

### 6.1 Income liquidity

A critical issue in the intersection of health and income research is the social stratification that manifests in disparities between individual health and income levels. This stratification often results in significant health disadvantages for groups with lower economic and social status, leading to health inequality (61, 62). To delve deeper into the income effects of health investment, we turn to income mobility indicators, which capture shifts in income classes. A decline in income mobility can ossify the social structure and impede the upward mobility of middle and low-income groups (63).

The income transition matrix is an essential tool for analyzing income mobility (64). At its heart, the matrix evaluates the likelihood of individuals transitioning between different income groups over a given period. Building on prior studies, our income matrix employs a decile approach, ranking income levels from 1 (lowest) to 10 (highest) and dividing them by quantiles. The foundational formula of the income transition matrix is as follows:


(4) $$P\left(x,y\right)=\left[p_{ij}\left(x,y\right)\right]\in R^{m\times m}$$

In the model (Eq. 4), $p_{ij}\left(x,y\right)$ represents the probability of an individual transitioning from grade I to grade J in the t-1 period, m is the number of grades arranged by income level from low to high, where 10 is taken, x and y represent the income levels at the beginning and end of the period, respectively,

We establish a cross period conversion matrix: m×m ^3^


(5) $$p=\left[\begin{array}{ccc} p_{11} & \cdots & p_{1m}\\ \vdots & \ddots & \vdots \\ p_{m1} & \cdots & p_{mm} \end{array}\right]$$

Utilizing the established methodology for assessing the fluidity of income positioning, the results of the income transition matrix are computed and displayed in Table 9.

**Table 9:** Income transfer matrix.

	1	2	3	4	5	6	7	8	9	10
1	16.12	25.47	20.7	15.32	10.45	4.98	2.79	2.09	1	1.09
2	8.21	17.32	23.12	19.72	15.12	7.71	4.9	1.6	1	1.3
3	3.75	10.21	15.73	20.83	19.17	14.27	8.96	3.33	1.98	1.77
4	2.83	5.47	8	18.12	21.46	18.52	13.66	6.78	2.63	2.53
5	1.69	3.38	5.17	6.95	17.39	22.37	20.49	12.97	5.83	3.76
6	2.31	2.69	3.06	2.41	7.13	17.41	24.72	21.3	12.04	6.94
7	1.49	2.28	1.29	2.38	2.67	8.02	18.02	29.5	24.65	9.7
8	0.58	0.23	0.46	0.58	1.04	2.78	4.87	23.52	41.25	24.68
9	0	0.61	0.15	0.45	0.45	0.91	1.82	6.96	35.1	53.56
10	0.26	0	0	1.02	1.79	3.07	3.58	5.37	16.62	68.29

Table 9 reveals that, on the main diagonal of both matrices, the values are the column maxima, signifying that the majority of the samples are most likely to maintain their income levels throughout the observation period. The increasing values along the diagonal suggest a trend toward greater stability at each level, with income mobility decreasing–particularly for individuals at higher income levels, where the tendency for income to become entrenched is more pronounced compared to those in low and middle-income brackets.

### 6.2 Health investment and income fluidity

In line with the scholarly framework, this article employs two metrics to gage income fluidity: (1) The variable of annual household income position change and mobility, which are discrete variables. To clearly delineate the dynamics of income mobility–whether ascending or descending–values of [−1, 0, 1] are assigned to represent downward mobility, stasis, and upward mobility, respectively, thereby capturing the direction of residents’ income level shifts (65); (2) The overall shift in household income position is quantified by the difference between the end-of-period and initial income positions for a single sample.

Subsequently, we investigate the effect of health investments on the annual variation in household income position and the aggregate shift in household income position. Given the inherent ordered nature of income fluidity, which is part of an ordinal series, the ordered Probit model is utilized to analyze the impact of health investment on income fluidity (Table 10).

**Table 10:** Impact of health investment on income liquidity.

variable	Dependent variable: income change	
Score	0.051	0.420***
	(0.248)	(2.707)
Control variable	Join	Join
Year fixed	Join	Join
Family fixed	Join	Join
*N*	3,305	3,863

The values in parentheses are *t*-values, where *represents a 10% significance level, **represents a 5% significance level, and ***represents a 1% significance level.

The data from Table 8 indicates that health investment has a statistically significant effect on the overall shift in household income position at a 1% confidence level. This suggests that health investment can markedly enhance the overall change in household income position. However, it appears that health investment does not have a significant impact on the annual variation in household income position.

### 6.3 The impact of health investment on Shorrocks value

Utilizing the income transition matrix, we apply the Shorrocks indicator to gage income fluidity. The Shorrocks indicator, predicated on a set of assumptions, quantifies the influence of past income levels on current ones. This measure can not only ascertain whether the relative income position of a sample has shifted over the long term but can also gage the magnitude of such changes. The foundational formula for the Shorrocks indicator is as follows:


(6) $$M\left(p\right)=\frac{m-\sum_{i=1}^{m}p_{ii}}{m-1}$$

In the model (6), m represents the income level^4^ and $\overset{m}{\underset{i=1}{\sum }}p_{ii}$ represents the trace of the income conversion matrix, which is the sum of the diagonal elements of the conversion matrix (Table 11).^5^

**Table 11:** Health investment and Shorrocks indicators.

	Full sample		Low Income sample	
variable	One stage regression (Dependent variable: score)	Two stage regression (Dependent variable: Shorrocks)	One stage regression (Dependent variable: score)	Two stage regression (Dependent variable: Shorrocks)
Dietary	0.017**		0.016**	
	(2.182)		(1.969)	
Score		0.021		0.048***
		(1.566)		(3.052)
control variable	Join	Join	Join	Join
Year fixed	Join	Join	Join	Join
Family fixed	Join	Join	Join	Join
*N*	3,334	3,334	2,235	2,235

The values in parentheses are *t*-values, where *represents a 10% significance level, **represents a 5% significance level, and ***represents a 1% significance level.

Upon calculating the Shorrocks index for each household, the study employs a benchmark model to assess the influence of health investment on this index. The findings reported in Table 9 indicate a positive yet statistically insignificant impact of health investment on the Shorrocks index for the overall sample. However, for samples from low-income households, the effect of health investment on the Shorrocks index is both statistically significant and positive at the 1% confidence level. This reveals that health investment notably influences income mobility for lower-income households.

### 6.4 Distinguishing income growth from income gap reduction

In examining the effects of health investment, it is crucial to differentiate between income growth and income gap reduction. This paper investigates these two outcomes as separate explanatory variables. Income growth is quantified by the proportionate change in income from 1 year to the next, specifically, (current year’s income - previous year’s income) divided by the current year’s income (64). The income gap, on the other hand, is evaluated using the expenditure Gini coefficient at the district and county level (66), providing a measure of the income disparity within these areas (Table 12).

**Table 12:** Income effect of health investment: income growth or income gap reduction.

Variable	One stage regression (Dependent variable: score)	Two stage regression (Dependent variable: income change)	One stage regression (Dependent variable: score)	Two stage regression (Dependent variable: income disparity)
Dietary	0.025**		0.017	
	(2.194)		(1.535)	
Score		0.296		−4.745*
		(0.194)		(−1.876)
Control variable	Join	Join	Join	Join
Year fixed	Join	Join	Join	Join
Family fixed	Join	Join	Join	Join
*N*	1943	1943	2,270	2,270

The values in parentheses are *t*-values, where *represents a 10% significance level, **represents a 5% significance level, and ***represents a 1% significance level.

The analysis presented in Table 10 reveals that while health investment positively influences income growth, the effect is not statistically significant. However, the impact on the income gap is significant, showing a negative correlation at a 10% confidence level. This suggests that health investment contributes more to narrowing the income gap than to fostering income growth.

## 7 Conclusion and further research

Drawing on data from the China Nutrition and Health Survey (CHNS) spanning 1989 to 2015, this study concludes that health investment significantly boosts both absolute and relative income. The income effects of health investment differ across urban and rural areas, regions, and professions, with mechanisms including illness duration, employment, effective labor time, and education. Robustness tests reveal that the income effect of health diminishes with increasing income levels, yet the impact of health investment on income is sustained over time. Further examination shows that health investment substantially enhances the overall shift in household income position, particularly affecting income mobility in low-income households, and is more instrumental in reducing the income gap, thus benefiting low-and middle-income groups the most.

Future research should focus on two aspects:

Firstly, while the study provides valuable insights into the relationship between health investment and income within the Chinese context, it is essential to recognize the potential limitations in generalizing these findings to other countries with different socio-economic and health systems. The unique socio-economic landscape and healthcare infrastructure of China may influence the dynamics between health investment and income in ways that may not be directly applicable to other countries. Therefore, caution must be exercised when extrapolating the results of this study to different global contexts. Future research should explore similar relationships in diverse socio-economic and health system contexts to enhance the generalizability of findings and inform global health policy efforts effectively.

Secondly, China has implemented healthcare reforms aimed at improving access to services and promoting health equity since 2015, which may have influenced health investment behaviors and income distribution patterns. Additionally, shifts in socioeconomic factors, such as changes in income levels and employment structures, may have further shaped these dynamics. Moreover, advancements in technology, demographic changes, and other contextual factors contribute to the evolving landscape of health investment and income distribution. By synthesizing historical trends with current knowledge, we can anticipate the impact of recent trends or policy changes on health investment and income distribution, informing future research and policy interventions aimed at promoting equitable economic welfare in China. Recent healthcare reforms aimed at improving access to services and promoting health equity are likely to incentivize greater health investment among the population, particularly in preventive care and chronic disease management. This could lead to improved health outcomes and potentially contribute to higher income levels, as healthier individuals may be more productive in the workforce. Additionally, shifts in socioeconomic factors, such as changes in income inequality and employment structures, may influence patterns of health investment and income distribution. For example, efforts to address income disparities through social welfare programs or minimum wage policies could impact household income distribution and, consequently, affect individuals’ ability to invest in their health. Moreover, advancements in technology, such as the widespread adoption of telemedicine and health monitoring devices, may facilitate greater access to healthcare services and empower individuals to make informed health investment decisions. Overall, recent trends and policy changes are likely to have complex and multifaceted effects on health investment and income distribution in China, necessitating ongoing research and policy evaluation to ensure equitable economic welfare for all segments of the population.

## Data availability statement

Publicly available datasets were analyzed in this study. This data can be found at: https://opendata.pku.edu.cn/dataverse/CHADS.

## Author contributions

LZ: Formal analysis, Funding acquisition, Methodology, Project administration, Writing – original draft. WH: Writing – review & editing. MH: Writing – review & editing.

## Footnotes

^1^Determine whether the sample is a retirement sample through two questions in the CHNS questionnaire: “Why are you unemployed?” and “Are you retiring and reemployed.

^2^Dietary knowledge, “Do you know the Chinese Dietary Pagoda or Dietary Guidelines for Chinese Residents?” The CHNS database has been conducting surveys on respondents’ understanding of dietary knowledge since 2004 as a dummy variable.

^3^This matrix is a double random matrix, where the sum of elements in each row and column is 1. The larger the elements on the main diagonal, the greater the probability that individuals in a certain income level in the previous period will remain unchanged in the current period, and the lower the income liquidity.

^4^Each rank contains the same number of members.

^5^The smaller the trace of the transformation matrix, the higher the Shorrocks value, indicating that members in a certain income level in the previous period have a lower probability of remaining in that income level in the current period, and a higher probability of turnover.
